# Modeling the Differences in Biochemical Capabilities of *Pseudomonas* Species by Flux Balance Analysis: How Good Are Genome-Scale Metabolic Networks at Predicting the Differences?

**DOI:** 10.1155/2014/416289

**Published:** 2014-02-24

**Authors:** Parizad Babaei, Tahereh Ghasemi-Kahrizsangi, Sayed-Amir Marashi

**Affiliations:** Department of Biotechnology, College of Science, University of Tehran, Tehran 1417614411, Iran

## Abstract

To date, several genome-scale metabolic networks have been reconstructed. These models cover a wide range of organisms, from bacteria to human. Such models have provided us with a framework for systematic analysis of metabolism. However, little effort has been put towards comparing biochemical capabilities of closely related species using their metabolic models. The accuracy of a model is highly dependent on the reconstruction process, as some errors may be included in the model during reconstruction. In this study, we investigated the ability of three *Pseudomonas* metabolic models to predict the biochemical differences, namely, iMO1086, iJP962, and iSB1139, which are related to *P. aeruginosa* PAO1, *P. putida *KT2440, and *P. fluorescens* SBW25, respectively. We did a comprehensive literature search for previous works containing biochemically distinguishable traits over these species. Amongst more than 1700 articles, we chose a subset of them which included experimental results suitable for *in silico* simulation. By simulating the conditions provided in the actual biological experiment, we performed case-dependent tests to compare the *in silico* results to the biological ones. We found out that iMO1086 and iJP962 were able to predict the experimental data and were much more accurate than iSB1139.

## 1. Introduction

### 1.1. Metabolic Networks

Recent advances in sequencing techniques have accelerated the process of whole genome sequencing of different organisms [1–3]. Additionally, computational tools for genome annotation have improved widely, which leads to better understanding of the function of genomic sequences [4, 5]. With this information in hand and a large amount of data about biochemical pathways accessible in various databases and scientific literature, the opportunity of emergence for the genome-scale metabolic networks has been provided in the last decade [6]. Such metabolic network models provide us with the opportunity to explore the physiological properties of different microorganisms with biotechnological applications [7].

### 1.2. Reconstruction and Mathematical Modeling of Metabolism

A typical framework for modeling cellular metabolism is constraint-based modeling, which involves stoichiometric and reversibility constraints on reactions [7, 8]. For a particular organism, considering cellular metabolites as the rows and metabolic reactions as the columns of the stoichiometric matrix, a metabolic network model is created in an iterative process. The element *S*_*ij*_ of this matrix is the stoichiometric coefficient of metabolite *i* in reaction *j*, considering positive values for products and negative values for reactants. Each (enzymatic) reaction, in turn, is associated with one or a few metabolic genes. In fact, the process of metabolic network reconstruction involves compiling the metabolism of an organism to a machine-readable format. There are various ways to reconstruct a model ranging from bottom up, totally manual approach [9] to the semiautomated process such as rBioNet [10], GEMSiRV [11], and RAVEN [12]. Available genome-scale metabolic network models have a wide spectrum from bacteria [13–15], to archaea [16], and to mammals and human [17–20].

The common point of the all reconstruction processes is the iterative validation of the model. By performing cycles of *in silico *experiments and comparing them to the wet-lab results, the discrepancies of the model and the actual organism are recognized. Subsequent model refinements pave the way for having a reliable framework to perform *in silico* experiments. Such a model allows the researchers for an in-depth and systematic investigation of the cellular metabolism. In the field of genetic engineering [21, 22], drug targeting [23–25], and evolutionary studies [26–28], models have been very useful so far.

### 1.3. Goal of the Present Study

Despite the increasing number of available metabolic models, to the best of our knowledge, little effort has been put towards comparing biochemical capabilities of closely related species using their genome-scale metabolic models. Since there is no unique approach to reconstruct a network, the models may contain process-dependent errors (including missing reactions). Therefore, one should not expect the models to reflect the biochemically distinguishable differences as successful as the wet-lab results. Nevertheless, if a model is accurate enough, the *in silico* results should be close to the real-world data. In this study, we used three *Pseudomonas* models: iMO1086 for *Pseudomonas aeruginosa* PAO1 [29, 30], iJP962 for *Pseudomonas putida *KT2440 [30, 31], and iSB1139 for *Pseudomonas fluorescens *SBW25 [32] to interrogate the capability of models to reflect biological differences amongst these three *Pseudomonas* species.

## 2. Materials and Methods

### 2.1. Database Search

We decided to compare the models two by two. Considering this, three cases were to be investigated. In first step, we comprehensively searched PubMed database for the entries containing two of the three *Pseudomonas* species. Primary search results included 675 articles for *aeruginosa-putida* case, 456 articles for *fluorescence-putida* case, and 622 articles for *aeruginosa-fluorescence* case.

The abstracts of these articles were carefully reviewed in order to select the articles of interest. The selected articles in this step were those articles in which biochemically distinguishable differences between the two species were investigated. These articles were reviewed again in detail by reading their full-texts thoroughly, in order to see whether they fulfill the selection criteria (see below).

### 2.2. Biochemical Properties Which Can Be Verified *In Silico*

Not every wet-lab experiment is suitable to be simulated *in silico*. The first criterion is that the particular pathway/gene/enzyme/reaction discussed in the article should be included in the model, as we are not supposed to change the structure of the model. In other words, the goal of this study is to understand the potential of the available genome-scale metabolic networks to model the biochemical differences, not to present improved models. Flux through a reaction and the essentiality of a gene in a specific environmental condition could be tested for the models.

#### 2.2.1. Biochemical Differences

The three *Pseudomonas* species have a part of their metabolism in common. However, these shared pathways are not exactly the same. For example, the reactions might have different fluxes. On the other hand, some genes or pathways are specific to each of these species. Pathogenesis for *P. aeruginosa *[33, 34] and catabolic pathways of aromatic compounds for *P. putida *[35, 36] are the examples of this type of differences. The articles investigating this kind of biochemical differences are suitable for *in silico *simulation.

#### 2.2.2. Genetic Engineering

Genetic engineering procedures are favorable for *in silico *simulation. Any changes made in the experiment in order to investigate cellular response to a perturbation should be in the form of adding or removing a particular pathway/gene/enzyme/reaction, or otherwise it could not be tested. Modeling gene and pathway addition or deletion is possible with COBRA toolbox [8, 37] (see below).

#### 2.2.3. Cellular Response Interrogation to Environmental Perturbations

Any metabolic model is a biochemical system with boundaries defined by exchange reactions. These reactions allow the substances to enter or leave the system. Thus, the experiments which have studied the cellular behavior in a particular growth medium or growth conditions could be computationally simulated if the related exchange reactions are included in the model. For instance, the experiments including measuring growth yield on different carbon or nitrogen sources could be simulated by setting the lower and upper bounds of the related exchange reaction to the same constant value. Besides, the upper bound and the lower bound of the other competing exchange reactions should be set to zero. Therefore, no carbon/nitrogen source but the ones provided by the medium could enter the cell. Finally, tolerance to a specific substance is also of interest, since by increasing the related reaction bound and performing FBA, the cellular response to an environmental tension could be simulated and compared to the actual results.

The articles that performed one of the above types of experiments for at least two of the species were ultimately selected to be the reference for our simulations.

### 2.3. Flux Balance Analysis (FBA)

FBA is a computational method for calculating the flux distribution of a metabolic network under a specified condition [38]. FBA optimizes an objective function (e.g., growth rate) based on the steady-state assumption, which states that the concentration of the cellular metabolites should not vary during the optimization process. This assumption is applied by considering *S* · **v** = 0, whereas *S* is the stoichiometric matrix and **v** is the flux distribution vector. Furthermore, each flux distribution **v** is also constrained by the “capacity” of reactions in the form **a** ≤ **v** ≤ **b**. For example, irreversible reactions *i* can only carry nonnegative flux values; that is, 0 ≤ *v*_*i*_.

As mentioned, in FBA flux through a linear biomass-producing reaction is maximized, based on the evolutionary assumption that a cell tends to maximize its growth. The biomass reaction includes the substrates needed for reproduction with appropriate stoichiometric coefficients, which are based on the ratios of the components forming the cellular dry weight and the energy needed for cellular maintenance (ATP hydrolysis).

### 2.4. COBRA Toolbox

We used COBRA toolbox to simulate the wet-lab experiment conditions. This toolbox provides us with various functions to perform *in silico *tests. Firstly, we modeled the experimental conditions, and then FBA was performed to see the model growth yield and flux distribution in the specified conditions.

For genetic engineering experiments, these two functions of the COBRA toolbox were used: “addReaction” and “removeRxns.”

- “addReaction” receives the chemical equation of the reaction and adds it to the model. If the metabolites are new to the model, they are inserted automatically as a new row to the *S* matrix.
- “removeRxns” gets the reaction IDs and deletes them by setting the lower bound and the upper bound of the cited reaction to zero.

In order to simulate the environmental conditions, exchange reaction should be altered manually. The function “changeRxnBounds” was used for this purpose. It can be used to alter either lower or upper bound of a specific reaction.

After modeling the experimental conditions, using COBRA toolbox functions, we ran FBA to see how the model responds and whether it is comparable to the wet-lab results.

## 3. Results and Discussion

### 3.1. Growth on Benzene under Different Oxygen Regimes

The growth patterns of the three *Pseudomonas* species during benzene degradation have been previously investigated under different oxygen regimes, namely, oxic, hypoxic, and anoxic regimes [39]. All of the strains used in the experiment were capable of growing under all oxygen regimes but in a different manner. All strains were inoculated in mineral salt medium containing aqueous benzene. MSM medium contains the following salts: K_2_HPO_4_, KH_2_PO_4_, (NH_4_)_2_SO_4_, MgCl_2_, CaCl_2_, H_3_BO_3_, ZnSO_4_, NiSO_4_, (NH_4_)_6_Mo_7_O_24_·4H_2_O, CuSO_4_·5H_2_O, MnSO_4_, CoCl_2_, and FeCl_3._

To define the *in silico *medium, the “exchange reactions” in iMO1086 and iJP962 and “uptake reactions” in iSB1139 were used. Since not all the ions used in the wet-lab medium were included in the models, it was just possible for the ions which had an associated reaction to enter the model. There was no uptake reaction for benzene in the model. Therefore, we used benzoate as the sole carbon source in our simulations. Benzene and benzoate are both converted to catechol in the cell [40, 41]. Hence, it is reasonable to use benzoate instead of benzene. For carbon source, we set the lower bound and upper bound of all possible carbon source exchange/uptake reactions to zero, except for benzoate. The default maximum flux for the uptake of carbon source for iMO1086 and iJP962 is 10 mmol·gDW^−1^·hr^−1^. We used this upper bound value as a constraint for the all carbon sources used in this study. For simulating oxic, hypoxic, and anoxic conditions, we gradually decreased the maximum possible rate of oxygen uptake. We considered the possible oxygen uptake flux to be greater than 50 mmol·gDW^−1^·hr^−1^ as the oxic, between 10 mmol·gDW^−1^·hr^−1^ and 50 mmol·gDW^−1^·hr^−1^ as hypoxic, and below 10 mmol·gDW^−1^·hr^−1^ as anoxic condition.

**Figure 1:**
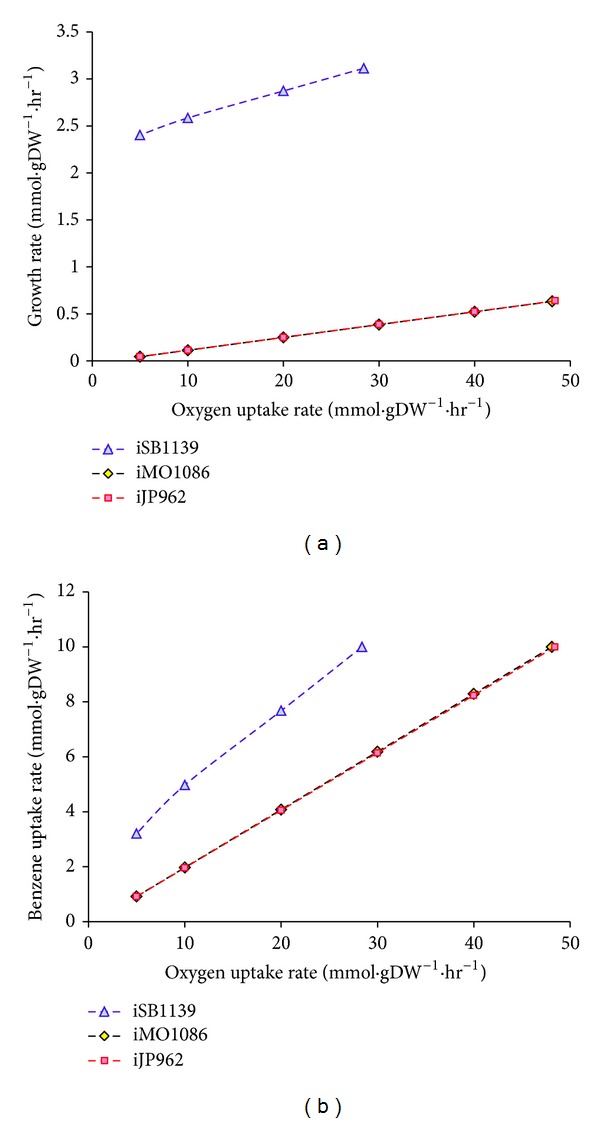
(a) *In silico* modeling of *Pseudomonas* species growth on benzoate under different oxygen regimes. In all three cases, biomass production increases as the oxygen uptake flux increases. The same pattern exists in all of the models. However, experimental data suggested different trends of response to the available environmental oxygen. (b) Benzoate uptake flux under different oxygen regimes. Similar to the growth rate, flux of the uptake reaction increases as the available environmental oxygen concentration augments in contrast to the experimental data.

Wet-lab experiments [39] showed that* P. aeruginosa* grew best under oxic conditions and cell growth decreased as the conditions changed from oxic to hypoxic. This decrease continues as the oxygen regime changes from hypoxic to anoxic. Therefore, cell growth is dependent on oxygen availability. Similar to the cell growth, benzene degradation pattern was highly dependent on environmental oxygen. By simulating the carbon source in the medium and oxygen regimes *in silico, *we ran FBA to see how the model would respond to the above-mentioned conditions. Results are summarized in Figure 1. By lowering the flux of oxygen exchange reaction, biomass production rate and flux through benzene degradation pathway decreased.

**Figure 2:**
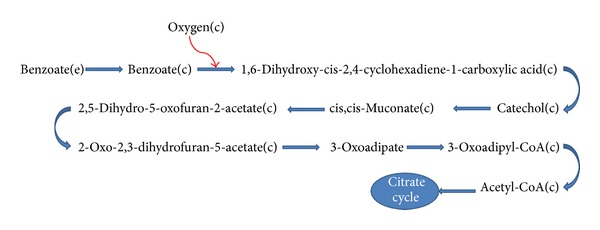
Benzoate degradation pathway. As depicted in the map, after benzoate uptake, cytoplasmic benzoate is converted to 1,6-dihydroxy-cis-2,4-cyclohexadiene-1-carboxylic. Since oxygen is one of the reactants, lowering the flux of oxygen uptake would in turn decrease the flux of this reaction, and the whole pathway would be less active, carrying lower flux values. This map shows only a part of metabolism.

Experimental results for *P. putida *and *P. fluorescens *were different from those of *P. aeruginosa.* The highest cell growth of *P. putida* and *P. fluorescens* were achieved under hypoxic and anoxic conditions, respectively. On the other hand, *P. putida* KT2440, the strain used for computational modeling, is known to be a strict aerobe [42], which is definitely different from the strain used to obtain the experimental data. Thus, we do not expect to see a consistency in this case. Simulating this experiment for iJP962, we got almost the same results as iMO1086. Besides, by performing the *in silico *experiment of growth on benzene for iSB1139, we obtained qualitatively the same results as the case of *P. aeruginosa.* This implies that the benzene catabolism between these three models might be the same. We decided to look more carefully into this pathway (see Figure 2). All of the three networks metabolize benzene through the same aerobic process in computational simulations and therefore it is natural to see almost the same results in all three cases. Although wet-lab experiments show different growth patterns, in all of them, biomass and benzene degradation flux were dependent on oxygen regime in a same manner. This might be due to the difference in the strains used in the experiment with the ones for which the models are reconstructed. For example *P. putida* KT2440 is a strict aerobe [42], whereas the *P. putida* strain used in the wet-lab experiments grew anaerobically. Another reason is that the benzene degradation pathway used in all of the models is the same and needs oxygen in the first step. Hence, regardless of the different structure of the models, oxygen flux is the determinant factor of benzene degradation pathway flux.

### 3.2. Growth Inhibition by Benzene

It has been shown that high benzene concentration in a growth medium can have toxic effects on the cells. In a wet-lab study, different *Pseudomonas* species have been grown under high benzene concentrations in MSM medium in order to investigate the tolerance of these species to benzene [43]. Initial inhibition of cell growth occurred in *P. putida* when aqueous benzene concentration was increased to 200 mg/L, while* P. fluorescens *stopped growing when benzene concentration approached 300 mg/L. The highest tolerance was 400 mg/L for *P. aeruginosa.*

**Figure 3:**
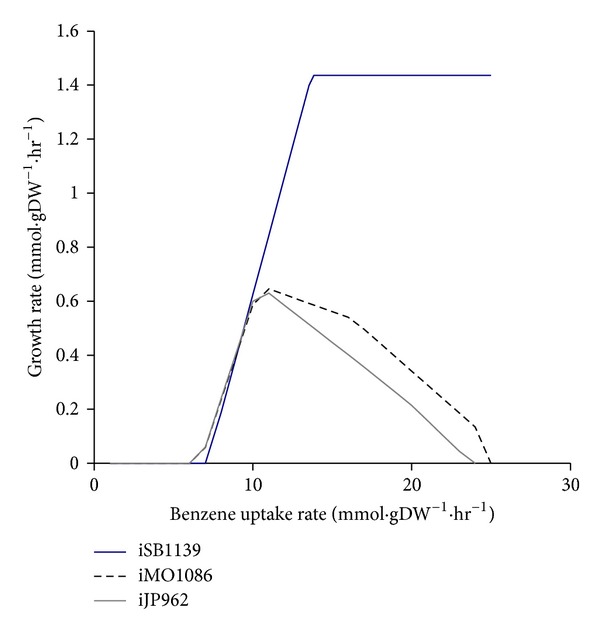
*In silico* modeling of *Pseudomonas* species growth on benzoate in MSM medium with a fixed oxygen uptake flux of 50 mmol·gDW^−1^·hr^−1^. Growth inhibition by high uptake fluxes of benzoate is seen in iMO1086 and iJP962. However, for iSB1139, the growth rate did not decrease, even at high benzene concentrations.

By performing *in silico* simulation of MSM medium with benzoate as the sole carbon source and fixing the oxygen exchange flux rate on 50 mmol·gDW^−1^·hr^−1^, we ran FBA to see how biomass production changes in response to increasing benzoate uptake flux. Results are shown in Figure 3. The biomass flux reached its maximum when benzoate uptake was 11 mmol·gDW^−1^·hr^−1^ for both iMO1086 and iJP962. After the peak, by increasing benzoate uptake flux, biomass flux decreased gradually to zero. The maximum possible flux for benzoate uptake reaction was 24 mmol·gDW^−1^·hr^−1^ for iMO1086 and 23 mmol·gDW^−1^·hr^−1^ for iJP962. For iSB1139, we did not see any decrease in growth rate. The growth rate initially increased as the benzoate uptake flux increased. However, for benzoate uptake fluxes greater than 13.84 mmol·gDW^−1^·hr^−1^, the growth rate reaches the constant value of 1.43 mmol·gDW^−1^·hr^−1^.

The computational metabolic models of *P. aeruginosa* and *P. putida* successfully reproduced the benzene inhibition effect, although they were not successful in explaining the differences in the tolerance levels of the two species. On the other hand, iSB1139 failed to simulate the growth inhibition by high concentration of benzene, which might be due to the “loose” structure of the model, existing internal loops, or exchange reactions which let benzoate or its metabolized forms exit the metabolic system.

### 3.3. Catabolism of Arginine

There are various pathways in different bacteria which enable them to utilize arginine as the sole source of carbon and nitrogen [44]. A previous study was conducted to determine the relationships between ornithine, citrulline, and arginine utilization pathways in *Pseudomonas* and related bacteria. The doubling time of the various strains including *P. aeruginosa* PAO1, *P. fluorescens* ATCC 13525, and *P. putida* IRC 204 on minimal medium containing arginine, succinylarginine, ornithine, or citrulline as carbon and nitrogen source has been measured in this study [45].

We used the models of *P. aeruginosa*, *P. fluorescens,* and *P. putida* to predict the biomass production rates in the experimental conditions of the study. For *in silico *simulation of growth on arginine or succinate or ornithine as the sole source of carbon and nitrogen, we set the flux rate of glucose and ammonium to zero and flux rate of desired metabolite's uptake/exchange reaction to 10 mmol·gDW^−1^·hr^−1^. Neither Succinylarginine nor citrulline uptake reactions are included in models, nor the catabolic pathways of these compounds. Therefore, these two cases were ruled out in our study. Results are shown in Table 1.

The cell growth was measured by the doubling time of each strain. Growth rate is associated with the inverse of the doubling time, and therefore we expected to see higher growth rates for shorter doubling times. From the *in silico* data in all three models, it can be seen that the biomass production rate increases in the cases where the model was allowed to use two carbon sources. When we let succinate and arginine enter the model, we are not able to force the model to use arginine only as the nitrogen source. In all three cases, the models were able to grow on arginine as the sole source of carbon and nitrogen. Allowing the models to use another carbon source like succinate would increase the biomass production rate. The same fact was seen in case of ornithine and succinate. When succinate was used as the carbon and ammonium as the nitrogen source, NH_4_^+^ was only the nitrogen source and could not be used as the carbon source as well. Therefore, one should compare the results in a way that the experiments having a single carbon source are compared to each other and not to the ones that have two potential carbon sources.

By comparing the growth rates of iMO1086 and iJP962, we concluded that these two models were almost consistent with experimental data, except for the case of succinate and arginine. In other words, since the doubling time of *P. putida* is greater than that of *P. aeruginosa*, one should expect that the growth rate of *P. putida* is less than *P. aeruginosa*, which is not the case in the simulations. In case of ornithine as the sole source of carbon and nitrogen, the differences between the doubling times were more than what we observed in the computational results.

For *P. fluorescens* in all cases, the biomass flux was higher than that of the other two models. This might be because of the “loose” (less-constrained) nature of the iSB1139. Comparison of the results of this model under different conditions could be more reliable. However, by doing so, the growth rate values predicted by the model are far from the experimental data. It suggests that this model needs some refinements and additional constraints, and it may overestimate the biological characteristics.

### 3.4. d-Fructose Catabolism

To investigate the pathways of d-fructose catabolism in *Pseudomonas* species, specific activities of the fructose, and succinate catabolism enzymes in cell-free extracts of *P. putida*, *P. fluorescens, *and *P. aeruginosa, *and also the doubling time of these species have been measured. Failure of *edd* ^*−*^ mutant of *P. putida* in Entner-Doudoroff pathway was also shown, while this mutant strain was able to grow on d-fructose [46].

In the experiment, bacteria were grown on Palleroni-Doudoroff liquid medium [47], containing either d-fructose or succinate. Therefore, in our simulations, we let the model consume ammonium, chloride, magnesium, ferric ion, and citrate which are the components of this medium. In two sets of separate simulations, either fructose or succinate was set as the sole source of carbon and nitrogen. We further simulated the *edd* ^*−*^ mutant by deleting *edd* gene (PP_1010) from the model. The growth simulations on both fructose and succinate are shown in Table 2.

Similar to the previous test, succinate could not support biomass production as much as other carbon sources, as in all cases, growth on succinate had the lowest biomass flux. For the growth on d-fructose, iMO1086 and iJP962 were found to be fairly consistent with the wet-lab data. However, difference between the doubling times is much higher than the difference between growth rates. The *in silico *growth rate of iSB1139 in this case is too high as well and not comparable to the other two models.

The computational model of *edd* ^*−*^ mutant in iJP962 could not reproduce the experimental results. In the wet-lab experiments, this mutation almost doubled the doubling time but did not have any effects on *in silico *growth. The reaction associated to this gene could not carry any flux during growth on fructose or glucose. Therefore, its removal would not have any effect on the biomass production flux.

### 3.5. Construction of Nitrate Respiring *Pseudomonas putida* Kt2440

*P. putida* is vastly used in biotechnology. However, a major drawback of this strain is its incapability to grow anaerobically. To overcome this disadvantage, a genetically engineered strain was recently constructed by introducing the denitrification pathway of *P. aeruginosa* [48]. This strain was not able to grow under anoxic conditions, but significant anaerobic survival compared to control strains was observed.

In the experiment, modified M9 medium was used. This medium contained Na_2_HPO_4_, KH_2_PO_4_, NaCl, NH_4_Cl, MgSO_4_, CaCl_2_, FeSO_4_·7H_2_O, succinate, and pH 6.8 supplemented with either KNO_3_ or NaNO_2_. One milliliter trace metal solution was added, which contained ZnSO_4_·6H_2_O, MnCl_2_·4H_2_O, CoSO_4_·7H_2_O, NiCl_2_·6H_2_O, CuCl_2_·2H_2_O, Na_2_MoO_4_·2H_2_O, and HCl. The exchange reactions of these salts (their related ions) were allowed to carry flux wherever a corresponding exchange reaction was in the model. Although the medium was simulated as much as possible, it could not be simulated precisely.

**Figure 4:**
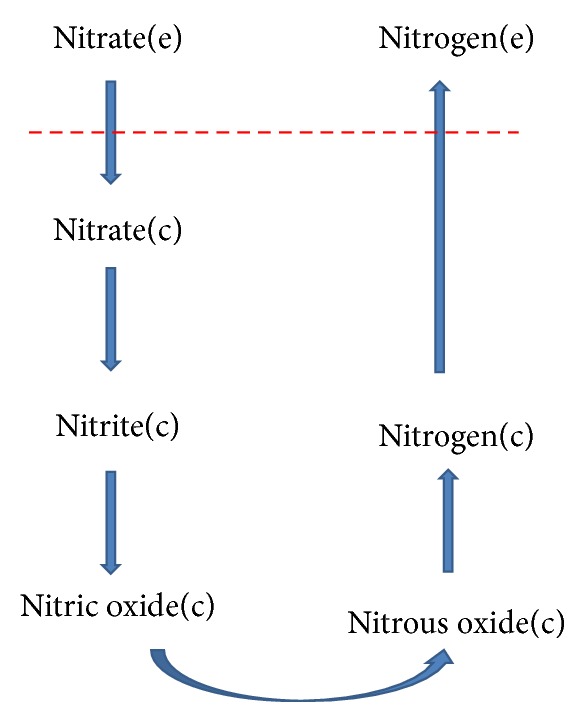
Denitrification pathway. Metabolites like water, proton, ferricytochrome *c*, ubiquinol, and ubiquinone are excluded for clarity. Dashed line shows the system boundary.

*In silico* simulation was performed by knocking in the reactions of denitrification pathway to iJP962. This pathway (Figure 4) begins with an exchange reaction which uptakes nitrate from medium. Then, the pathway continues with reactions that convert this metabolite to gaseous nitrogen (N_2_), which leaves the system.

By setting the flux of oxygen uptake reaction to zero and performing FBA, the model was able to have a possible non-zero flux distribution. However, the growth rate was zero in these conditions. Hence, the model did not show any growth in anaerobic conditions, similar to the genetically engineered strain in wet-lab. However, the *in silico* survival was increased as the network was able to consume nitrate. Thus, the model was able to reproduce the wet-lab results.

Adding new reactions to a model leads to some perturbations in the patterns of flux distributions. However, the denitrification pathway could function independent of the growth (i.e., even when the growth rate is zero), because none of the main metabolites of the pathway had been present in the model.

### 3.6. Growth on Leucine, Isovalerate, and Succinate

Catabolic pathway of saturated methyl-branched compounds like leucine and isovalerate has been previously investigated in *P. aeruginosa *and* P. putida* [49]. The gene products of leucine/isovalerate utilization pathway (Liu) have been studied in detail. The *liuRABCDE *gene cluster is essential for leucine/isovalerate utilization in *P. aeruginosa*. It is indicated that both species are able to grow on leucine or isovalerate as the sole carbon source. Both *P. aeruginosa *and* P. putida* grew well on isovalerate, while growth on leucine was more favorable for *P. putida *in comparison to *P. aeruginosa. In silico* result was able to predict this phenomenon, as the flux through biomass reaction of iJP962 was higher (although marginally) than that of iMO1086 (see below).

In this study, mineral salt medium was used with leucine or isovalerate as the sole carbon source. The components of this medium and *in silico* simulation of MSM are mentioned previously in the text. Here, for simulating growth on leucine/isovalerate, we set the lower bound and upper bound of the all exchange reactions of the all possible carbon sources to zero, except for leucine or isovalerate uptake reaction. According to the experimental results, *P. putida* could grow better than *P. aeruginosa* on leucine. They had the same growth pattern on isovalerate as the sole carbon source. Since the difference was related to growth on leucine, we simulated growth on this carbon source. *In silico *results were consistent with experimental data; that is, the growth rates of iJP962 and iMO1086 were 0.785 and 0.778 mmol·gDW^−1^·hr^−1^, respectively.

**Figure 5:**
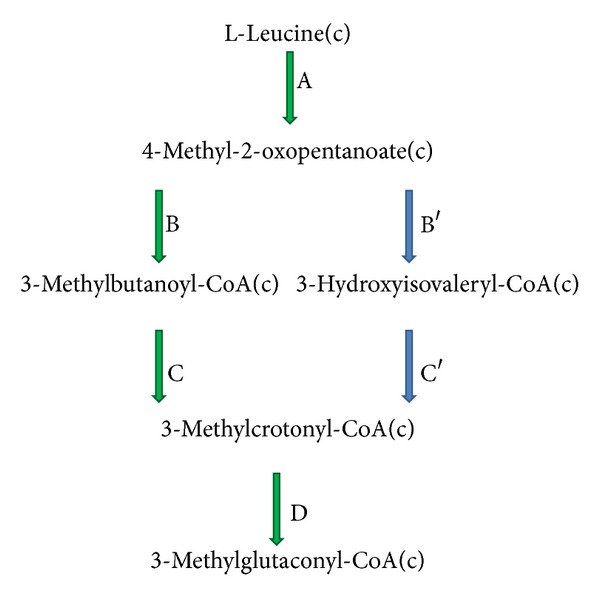
Leucine degradation pathway. The path in which the arrows are green is present in the model, while the path which includes 3-hydroxyisovaleryl-CoA is suggested by the experimental data.

In the above-mentioned experimental study [49], some mutations were made in order to determine the essentiality of each gene product in the pathway in *P. aeruginosa. *It appeared that by making insertion mutations in *liuC* and *liuD* genes, the mutants lost their ability to grow on leucine. Inactivation of *liuC *gene in iMO1086 had no effect on *in silico* growth, while removing the *liuD* gene led to zero flux in biomass production. We investigated the reason of this discrepancy and found out that the Liu pathway of the model was different from the one described in the experiment.  Figure 5 depicts a part of leucine metabolism. The pathway, which goes via 3-methylbutanoyl-CoA is included in the model, and the alternative which goes via 3-Hydroxyisovaleryl-CoA is the one suggested previously [49]. Reaction C′, which is related to *liuC*, is not utilized by the model. We added the reaction B′ to the model, upstream to C′, in order to complete the suggested pathway by the reference. The model did not include this reaction, although adding the new reaction did not change the preference of the model to carry flux through the pathway which goes via 3-methylbutanoyl-CoA. However, reaction D is associated with *liuD*. Since during growth on leucine, this reaction is active, its deletion led to zero growth rate. The strain used in this experiment and the strain which model is built for are the same (i.e., both are *P. aeruginosa *PAO1), and therefore the discrepancy could not be related to difference in the strains. We propose that such an inconsistency is presumably due to little or no flux in reactions B′ and C′ (if it is not an error in the reconstruction procedure and the bioinformatics data).

### 3.7. Utilization of l-Amino Acids as the Sole Source of Carbon and Nitrogen

According to several reports, amino acid metabolism might occur in bacteria under nutritionally poor conditions. Amino acids can serve as the sole source of carbon and nitrogen in complete absence of organic growth factors, and this physiological property is vastly seen amongst bacteria [50–52]. An experiment has been previously conducted to analyze the cell growth of two *Pseudomonas* species, namely, *P. fluorescens *and *P. aeruginosa *on single l-amino acids as sole source of carbon and nitrogen [53].

Lochhead-Chase basal medium [54] has been used with the modification that glucose and nitrate had been replaced by a single amino acid as both carbon and nitrogen source. Besides amino acids, this medium contained mineral salts. Since we could not determine the exact components of this medium, we performed the simulations in MSM medium which was also constituted of mineral salts. For making an amino acid, the sole source of carbon and nitrogen, we set the upper bound and lower bound of the exchange/uptake reaction all of the carbon and nitrogen sources to zero.

In the wet-lab experiment, the ability of growth on all twenty amino acids in the “l” form as the sole source of carbon and nitrogen was investigated. The results had been estimated visually and on a scale from 0 to 2, 0 meaning no growth and 2 meaning good growth. They also used 1 for poor growth. Seventeen of the amino acids produced almost the same growth response. Only l-threonine, l-tryptophan, and l-hydroxyproline had different effects on the cell growth pattern. Therefore, these data could be subject to our study since they reflect the biochemical differences.

We simulated the experiment *in silico* and performed FBA to see the biomass reaction flux and compare it to wet-lab results. *In silico* results are shown in Table 3. In the case of l-hydroxyproline, there was no uptake reaction in either of the models; hence, we could not perform FBA. We could add an exchange reaction to the model, keeping in mind that the consumed metabolite should be metabolized and a single uptake reaction may not be sufficient. Since the catabolic pathway of l-hydroxyproline is not included in the models, we did not perform the growth simulation on l-hydroxyproline.

For the case of l-threonine and l-tryptophan, computational results were not completely consistent with the wet-lab data. In iMO1086, cell growth occurred on l-threonine. *In silico* simulation in this case produced the same results. The model, however, was not able to grow on l-tryptophan in contrast to the wet-lab data. This inconsistency might be because of difference in the strain used in the experimental study or an error of the model.

In case of the iSB1139 model, growth on l-threonine was found to be possible. However, model could not produce any biomass on l-tryptophan. This could be due to the difference of experimental and *in silico *strains or missing pathways in the model.

### 3.8. Histidine Utilization By *Pseudomonas* Species

Amino acids like histidine are important sources of carbon, nitrogen, and energy for *Pseudomonas *species. Histidine degradation in bacteria occurs via either a four-step or a five-step enzymatic pathway [55]. *Pseudomonas fluorescens* SBW25 degrades histidine by the five-step pathway [56]. On the other hand, *Pseudomonas aeruginosa* PAO1 utilizes this amino acid through both four- and five-step pathways. Studies have shown that this bacterium does not necessarily use both routes at the same time and it can interchange the routes depending on the environment [57].

We assumed that by knocking out the five-step pathway in both species, *P. aeruginosa* would be able to grow on histidine, as it has the alternative histidine utilization pathway. However, the inactivation of the five-step pathway of *P. fluorescens* would lead to cell death on histidine, since the only catabolic pathway of this amino acid would be inactivated.

We simulated the minimal M9 medium conditions for both iMO1086 and iSB1139. For this purpose, we set the exchange reaction flux of glucose and ammonia to zero and instead we set the uptake rate of histidine to 10 mmol·gDW^−1^·hr^−1^.

**Figure 6:**
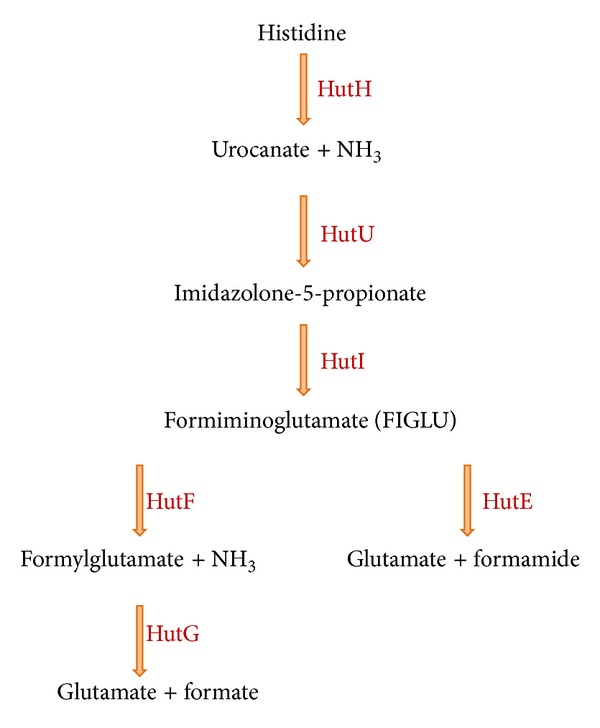
Histidine utilization pathway in *P. aeruginosa. *This strain can metabolize histidine either through a four-step or a five-step pathway. *P. fluorescens* is only capable of utilizing histidine through the five-step pathway.

As shown in Figure 6, the three first reactions are shared between these two pathways;  by blocking either *hutF* or *hutG,* the five-step pathway would be blocked. We simulated inactivation of the five-step pathway by inactivating *hutG* gene or its associated reaction. By deleting this gene, flux through this pathway did not change, as there was another gene associated with the reaction with “OR.” When we removed the reaction associated with *hutG*, the model could not grow on histidine. We investigated the reason of this inconsistency and we found out that only the five-step pathway of histidine utilization is included in iMO1086. Therefore, by deactivating the pathway, the model could not simulate the growth on histidine.

It should be noted that the model of *P. fluorescens*, that is, iSB1139, showed no growth on histidine as the sole source of carbon and nitrogen. These results are against the biological observations [56], which means that the model is presumably incomplete (if the disagreement is not related to the strain differences).

### 3.9. Amino Acid Utilization in *P. putida* versus *P. fluorescens*

Utilization of acidic amino acids and their amides as the sole source of carbon and nitrogen by *Pseudomonas *species has been investigated previously [58], and the growth profiles of *P. putida *and *P. fluorescens* have been studied qualitatively (Table 4). It has been shown that an enzyme involved in glutamate and aspartate utilization, called periplasmic glutaminase/asparaginase (PGA), is induced by glutamate and related amino acids. PGA is the product of *ansB* gene. It has also been shown that a mutant of *P. putida *KT2440 in *ansB* gene was not able to utilize glutamine, while growth on other amino acids was possible [58].

In the experiment, that is, growth on amino acids as the sole source of carbon and nitrogen, bacteria were grown on M9 minimal medium with amino acid as the sole source of carbon and nitrogen.

We have simulated the experimental conditions *in silico *by making the desired amino acid as the sole source of carbon and nitrogen and setting the upper and lower bound of the exchange/uptake reactions of all other possible carbon and nitrogen sources to zero.

Results of amino acid consumption are shown in Table 4. For *P. putida*, experimental and computational data were almost consistent. The only case of inconsistency was related to leucine in which no growth was seen in the wet-lab experiment, while the model produced a considerable flux rate of biomass production. Since the strain used in the experiment and *in silico *simulation was the same, this inconsistency might be due to falsely added reactions in the model.

For the model of* P. fluorescens*, there are several inconsistencies for cysteine, serine, glutamine, and glutamate. However, in this case, the strain used in the experiment (ATCC 13525) was different from the modeled strain (SBW25), which may (or may not) be the source of inconsistencies.

The lethal effect of *ansB* deletion for growth on glutamine has been indicated in this study. The product of this gene, PGA, is a periplasmic enzyme that converts glutamine to glutamate. Mutant cells lacking this enzyme were unable to grow on glutamine and concentration of glutamine in the medium did not decrease over time. Therefore, mutant cells were unable to uptake this amino acid. However, they could grow on glutamate and other amino acids [58].

By simulating experimental conditions, we performed gene essentiality analysis. Deleting *ansB* gene (PP0495) or removing its associated reaction had no effect on model growth. The reason of the inconsistency is that there are alternative pathways included in the model for converting glutamine to glutamate and hence these reactions performed the conversion in the absence of *ansB* product.

## 4. Concluding Remarks

To date, several genome-scale metabolic networks have been reconstructed. These models have been used for different purposes, ranging from basic science studies to metabolic engineering applications. However, to the best of our knowledge, using these models in a comparative manner has not attracted much attention previously.

In this study, we used three *Pseudomonas* metabolic network models in a series of *in silico* comparative simulations and then evaluated the computational results with the experimental data. It should be emphasized that for gathering information, we used the previously reported experimental data on the same species, but we did not limit our search necessarily to the strains for which the models were built. A number of studies have previously applied such a strategy for validating reconstructed metabolic network models [59–61]. However, this might lead to inconsistencies between experimental and computational results, as various strains of the same species have different biochemical properties. We encountered such a case in our study, namely, for the aerobic growth of *P. putida *on benzene (Section 3.1).

In our study, in most of the cases, *P. aeruginosa* and *P. putida *models could reproduce previously reported experimental results. However, the model for *P. fluorescens *failed to predict the biological data in several instances. One reason for high accuracy of iMO1086 and iJP962 is the vast availability of biological data for *P. aeruginosa* and *P. putida. *Since *P. aeruginosa *is an opportunistic human pathogen [33, 34] and *P. putida* is a bacterium of biotechnological importance [31, 48], the models of these strains include enough data after iterative refinement of the reconstruction process. Moreover, these two models have been further validated during a process called “reconciliation” [30], which includes comparing metabolic network models to eliminate errors that have been included in the model during the reconstruction.

The very recent* in silico* model of *P. fluorescens*, iSB1139, showed inconsistent results with experimental data in many of our simulations. It is known that the computational growth rate of this model is far from the reported experimental growth rate [32]. According to our results, this model needs further refinement in order to produce more reliable predictions.

## Figures and Tables

**Table 1:** Biological data and *in silico* results for arginine and succinate utilization of the bacteria and the models. Biological data represent doubling times and *in silico* results represent computational growth rates.

Additive(s) to minimal medium	*P. aeruginosa *		*P. putida *		*P. fluorescens *	
	*In silico *data (mmol·gDW^−1^·hr^−1^)	Biological data (min)	*In silico *data (mmol·gDW^−1^·hr^−1^)	Biological data (min)	*In silico *data (mmol·gDW^−1^·hr^−1^)	Biological data (min)
Succinate + NH_4_	0.397	60	0.401	54	0.933	66
Arginine	0.627	63	0.633	60	1.038	146
Succinate + arginine	0.933	46	1.057	52	2.076	48
Ornithine	0.606	240	0.616	90	1.038	420
Succinate + ornithine	1.020	86	1.034	56	2.076	390

**Table 2:** Growth rates of iMO1086, iJP962, and iSB1139 on fructose and succinate as the sole carbon sources. Biological data represent doubling times and *in silico* results represent computational growth rates.

Substrate	*P. aeruginosa *		*P. putida *		*P. putida *(*edd* ^−^)		*P. fluorescens *	
	*In silico *data (mmol·gDW^−1^·hr^−1^)	Biological data (min)	*In silico *data (mmol·gDW^−1^·hr^−1^)	Biological data (min)	*In silico *data (mmol·gDW^−1^·hr^-1^)	Biological data (min)	*In silico *data (mmol·gDW^−1^·hr^−1^)	Biological data (min)
Fructose	1.129	455	1.358	235	1.358	380	3.246	204
Succinate	0.980	73	0.980	53	0.980	52	2.296	59

**Table 3:** Relative cell growth estimated visually from amount of growth on amino acid.

Amino acid	*P. aeruginosa *		*P. fluorescens *	
	*In silico *data (mmol·gDW^−1^·hr^−1^)	Biological data	*In silico *data (mmol·gDW^−1^·hr^−1^)	Biological data
l-threonine	0.405	++	0.982	−
l-tryptophan	0	++	0	++
l-hydroxyproline	N/I	+	N/I	++

++: good growth; +: poor growth; −: no growth; N/I: uptake reaction not included in the model.

**Table 4:** Qualitative growth profiles of *P. putida* and *P. fluorescens* on three amino acids. Relative growth rates were estimated qualitatively.

Strains	*P. putida *KT2440		*P. fluorescens* ATCC 13525	
Amino acid as the sole source of carbon and nitrogen	*In silico *data (mmol·gDW^−1^·hr^−1^)	Biological data	*In silico *data (mmol·gDW^−1^·hr^−1^)	Biological data
Cys	0	−	0	+
Leu	0.785	−	0.156	(+)
Pro	0.639	++	0.161	(+)
Ser	0.283	+	0.964	++
Gln	0.538	++	1.246	+
GLu	0.556	++	1.244	+

−: no growth; (+): negligible; +: poor growth; ++: good growth.
